# Long‐term survival outcomes of patients with Niemann‐Pick disease type C receiving miglustat treatment: A large retrospective observational study

**DOI:** 10.1002/jimd.12245

**Published:** 2020-05-08

**Authors:** Marc C. Patterson, William S. Garver, Robert Giugliani, Jackie Imrie, Helena Jahnova, F John Meaney, Yann Nadjar, Marie T. Vanier, Patrick Moneuse, Olivier Morand, Daniel Rosenberg, Barbara Schwierin, Benedicte Héron

**Affiliations:** ^1^ Division of Child and Adolescent Neurology, Departments of Neurology, Pediatrics and Medical Genetics Mayo Clinic Rochester Minnesota USA; ^2^ Department of Chemistry and Chemical Biology University of New Mexico Albuquerque New Mexico USA; ^3^ Medical Genetics Service Porto Alegre Brazil; ^4^ Department of Genetics UFRGS Porto Alegre Brazil; ^5^ Niemann‐Pick UK Washington UK; ^6^ Department of Institute of Inherited Metabolic Disorders Charles University Prague Czech Republic; ^7^ Department of Pediatrics University of Arizona Tucson Arizona USA; ^8^ Department of Neurology Reference Center for Lysosomal Diseases (CRML), Hôpital de la Pitié‐Salpêtrière Paris France; ^9^ INSERM Lyon France; ^10^ Hopitaux de Lyon Lyon France; ^11^ Global Business and Science Affairs Actelion Pharmaceuticals Ltd. Allschwil Switzerland; ^12^ Epidemiology and Observational Studies, Actelion Pharmaceuticals Ltd., Allschwil Switzerland; ^13^ Azafaros B.V, Leiden The Netherlands; ^14^ Idorsia Pharmaceuticals Ltd. Allschwil Switzerland; ^15^ Department of Neuropediatrics, CRML, Hopital Armand‐Trousseau Paris France; ^16^ Sorbonne Universite Paris France; ^17^ Present address: Azafaros B.V Leiden The Netherlands; ^18^ Present address: Idorsia Pharmaceuticals Ltd. Allschwil Switzerland

**Keywords:** miglustat, Niemann‐pick disease type C, NP‐C, NPC registry, observational national cohorts, survival, Zavesca

## Abstract

Miglustat has been indicated for the treatment of Niemann‐Pick disease type C (NP‐C) since 2009. The aim of this observational study was to assess the effect of miglustat on long‐term survival of patients with NP‐C. Data for 789 patients from five large national cohorts and from the NPC Registry were collected and combined. Miglustat‐treated and untreated patients overall and within sub‐groups according to age‐at‐neurological‐onset, that is, early infantile‐onset (<2 years), late infantile‐onset (2 to <6 years), juvenile‐onset (6 to <15 years), and adolescent/adult‐onset (≥15 years) were analysed and compared. Survival was analysed from the time of first neurological manifestation (Neurological onset group, comprising 669 patients) and from diagnosis (Diagnosis group, comprising 590 patients) using a Cox proportional hazard model adjusted for various covariates. Overall, 384 (57.4%) patients in the Neurological onset group and 329 (55.8%) in the Diagnosis group were treated with miglustat. Miglustat treatment was associated with a significant reduction in risk of mortality in both groups (entire Neurological onset group, Hazard ratio [HR] = 0.51; entire Diagnosis group, HR = 0.44; both *P* < .001). The effect was observed consistently in all age‐at‐neurological‐onset sub‐groups (HRs = 0.3 to 0.7) and was statistically significant for late infantile‐onset patients in both groups (Neurological onset group, HR = 0.36, *P* < .05; Diagnosis group, HR = 0.32, *P* < .01), and juvenile‐onset patients in the Diagnosis group only (HR = 0.30, *P* < .05). Despite the limitations of the data that urge cautious interpretation, the findings are consistent with a beneficial effect of miglustat on survival in patients with NP‐C.

## Graphical abstract

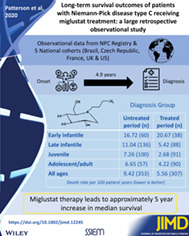

**SYNOPSIS**

This analysis of the largest cohort of patients with Niemann‐Pick disease Type C to date demonstrated that miglustat treatment improves the survival of patients suffering from this fatal neurodegenerative disease.

## 1 INTRODUCTION

Niemann‐Pick disease Type C (NP‐C) is a rare, neurodegenerative disease with an estimated incidence of 1:89000.^1^, ^2^, ^3^ It is caused by autosomal recessive mutations in *NPC1* (~95% of cases) or *NPC2*, which lead to intracellular accumulation of unesterified cholesterol and glycosphingolipids.^2^

NP‐C may present from the perinatal period until adulthood, with life expectancy spanning over seven decades.^2^ The clinical picture of NP‐C is extremely heterogeneous, usually presenting initially with visceral symptoms, and a small subset of patients with NP‐C die very early in life from severe systemic disease.^2^, ^3^ Systemic disease, when present, precedes neurological manifestations, which are progressive and ultimately result in death.^2^, ^3^ Non‐specific clinical presentation of NP‐C often prevents timely diagnosis, which has been shown to be delayed by a mean of 4.1 years from the onset of neurological manifestations.^3^ The age of neurological manifestation onset has been recognised as a predictor for severity, rate of disease progression, and life expectancy. Survival appears to be the poorest in patients with rapidly progressive early infantile neurological onset, whereas patients with more slowly progressive adult onset NP‐C tend to live longer.^2^, ^4^, ^5^, ^6^, ^7^

Miglustat (Zavesca, Actelion Pharmaceuticals), an iminosugar that inhibits glycosphingolipid synthesis, is currently the only approved disease‐modifying therapy for NP‐C. It has been in clinical use for the treatment of NP‐C for more than 10 years.^3^ Although several cohort studies and case series have reported miglustat use in clinical practice settings,^6^, ^8^, ^9^, ^10^, ^11^, ^12^, ^13^, ^14^, ^15^ there is a lack of longitudinal observational data on the effect of miglustat treatment on the overall survival of patients with NP‐C. As no single study had enrolled enough patients to conduct a comparative effectiveness analysis on survival, this exploratory study was performed in the largest pooled data set of real‐world, multinational data for patients with NP‐C. Its objective was to estimate the survival of patients with NP‐C treated with miglustat from the time of onset of their neurological manifestations, or from the time of their NP‐C diagnosis, and to compare it with patients not treated with miglustat.

## 2 METHODS

### 2.1 Study design

This was an exploratory analysis using existing observational data from the Actelion‐sponsored multinational NPC Registry (AC‐056C501), and five large national observational NP‐C cohorts (Brazil, Czech Republic, France, UK, and United States). The NPC Registry collected prospective data on incident and prevalent patients (n = 414; 2009‐2016) from 21 countries (EU and non‐EU countries, excluding US). The Brazilian observational cohort included retrospective data for incident patients (n = 51; 1991‐2015), the Czech observational cohort for incident and prevalent patients (n = 57; 1975‐2012),^16^ the French observational cohort for incident patients (n = 136; 1990‐2014),^13^, ^14^ and the UK observational cohort for incident and prevalent patients (n = 146; 1999‐2011).^4^ Relevant data were collected from patient medical charts, and in the French cohort, additionally from questionnaires sent to referral physicians. In the United States observational cohort,^5^ data for incident and prevalent patients (n = 88; 1975‐2002) were patient and/or family reported in response to a clinical questionnaire containing over 80 questions (Table S1).

For the NPC Registry, an Ethics Committee approval at all centres and written informed consent for all patients was collected. For the national cohorts, which already had local ethical approval (as reported below), no additional approval from an Ethics Committee and/or Institutional Review Board (IRB) was required for this secondary use of existing databases or medical charts. Patient privacy was ensured as no personal identifying information was transferred to Actelion. Brazil cohort: ethical approval number CAAE #4552 1115.0.0000.5327. Czech Republic cohort: all procedures were conducted in accordance with the ethical standards of the responsible committee on human experimentation (institutional and national) and the Helsinki Declaration of 1975, and 2000.^16^ The French study was approved by the Comité de Protection des Personnes, Ile de France VI.^14^ The UK cohort did not require ethical approval as it was decided by the Central Manchester Foundation Trust that the study was an audit.^4^ The US cohort was approved by the University of Arizona IRB through the Human Subjects Protection Program.^5^

### 2.2 Patient population

Patients included in the analyses were diagnosed with NP‐C, received standard medical care in a real‐world setting, and had data collected on the following variables: country of residence; sex; date of birth; date of and/or age at NP‐C diagnosis (for secondary outcome only); date of and/or age at onset of neurological manifestations (if date/age not available, it was imputed with the earliest date of available symptoms); date of enrolment for NPC Registry patients only; date of, or age at, death, or if patient had not died, date of, or age at, censoring/last follow‐up; miglustat treatment (ever or never treated with miglustat; if ever treated with miglustat, date of, or age at, earliest miglustat start). Patients were considered treated if they were ever treated with miglustat, and not treated if they were never treated with miglustat. As soon as patients started miglustat, they were considered treated until death or last follow‐up, regardless of treatment interruptions.

Patients were counted in both cohorts if they were not treated at the beginning of the follow‐up period and subsequently initiated treatment with miglustat; prior to initiation of miglustat treatment their data were included in the not‐treated group, and after miglustat initiation, in the treated group. Once a patient had been treated with miglustat, they could not return to the not‐treated group.

### 2.3 Study outcomes

The primary outcome was defined as the time from onset of neurological manifestations to death or censoring and was analysed on those patients for whom all the key variables, including date of and/or age at onset of neurological manifestations, were available or were derived from other available data (“Neurological onset group”). The secondary outcome was the time from diagnosis of NP‐C disease to death or censoring and was analysed on the “Diagnosis group” which included all patients in the Neurological onset group who additionally had a date of, and/or age at, diagnosis available. For both outcomes, data were analysed for the miglustat‐treated period and the miglustat‐untreated period (Figure S1).

### 2.4 Statistical analyses

Statistical analyses were exploratory. No sample size was calculated, and no minimum sample size was determined a priori. Unless otherwise stated, descriptive statistics were used to summarise continuous variables; categorical variables were summarised using the total number of available values and the number of available values in each category.

### 2.5 Main outcome

The NPC Registry and national cohort databases were pooled to form the overall pooled data set. Duplicate records for patients who were included in both the NPC Registry and one of the national cohorts were identified programmatically using probabilistic record linkage and paired.^17^ Where duplicate records were identified, they were manually curated to decide which of the duplicate records should be retained.

Heterogeneity was assessed using descriptive statistics of key variables from the pooled national cohorts and the NPC Registry, and by evaluating the homogeneity of variance for survival time between the NPC Registry and the pooled national cohorts for patients not treated with miglustat, using the Brown‐Forsythe test.

The main analysis consisted of an extended Cox model, which included treatment (miglustat‐treated period vs miglustat‐untreated period) as a time‐dependent covariate,^18^ and category of age at onset of neurological manifestations, country (each country in the NPC Registry and the national cohorts), and sex as covariates. Right truncation was applied to censor patients who were still alive at the end of the observation period; left truncation was applied to reduce immortal/survival bias of the NPC Registry data. Adjusted Kaplan‐Meier (KM) curves, adjusted hazard ratios (HR), the 95% confidence intervals (CI) and *P*‐values were calculated. The number of patients in the analysis, number (%) of patients who died, total patient‐years, and crude death rates (per 100 patient‐years; not adjusted for the key variables) were determined. Unadjusted HRs were also calculated.

Sub‐analyses were performed to estimate the survival of patients sub‐grouped according to age at onset of neurological manifestations: early infantile included patients with age at neurological onset <2 years; late infantile included patients with age at neurological onset of 2 to <6 years; juvenile included patients with age at neurological onset of 6 to <15 years; and adolescent/adult included patients with age at neurological onset ≥15 years.^19^ In addition, the proportion of miglustat treatment (%), defined as “duration of miglustat treatment (years) x 100 / survival time (years)”, was calculated for miglustat‐treated patients in all four age‐at‐neurological‐onset sub‐groups.

### 2.6 Additional analyses

Sensitivity analyses were performed in the primary model on the overall pooled data set by including additional covariates such as data source (the NPC Registry and national cohorts), era of diagnosis (miglustat pre‐approval era [before 2009] and post‐approval era [2009 and later]), and miglustat lag time (time between the onset of neurological manifestations and miglustat treatment initiation), in order to evaluate their influence.

## 3 RESULTS

### 3.1 Patient population

The NPC Registry provided data for 414 patients. The national cohorts provided data for 478 patients (Brazil, n = 51; Czech Republic, n = 57; France, n = 136; UK, n = 146; United States, n = 88) (Table S1). For the Czech Republic, UK, and US cohorts, the number of patients with NP‐C diagnosed prior to 1990 was 17, 10, and 25, respectively. After the removal of duplicate patients, the final pool included 789 patients; 669 patients were included in the Neurological onset group and 590 patients in the Diagnosis group (Figure S2). Overall, 384 (57.4%) and 329 (55.8%) in the Neurological onset and Diagnosis groups were treated with miglustat, respectively (Table 1). In general, miglustat was widely available from its approval for treatment of neurological manifestation of NP‐C in the European Union in January 2009 but some patients in the analysis received miglustat prior to this date, during clinical trials or off‐label in clinical practice.

**TABLE 1:** Patient characteristics

	Neurological onset group (N = 669)	Diagnosis group (N = 590)
**Male**, n (%)	330 (49.3)	292 (49.5)
Age at onset of neurological manifestations, years		
n	668	589
Mean (SD)	10.36 (10.5)	9.88 (10.2)
Median	6.1	6.0
Minimum, maximum	0.0, 60.5	0.0, 60.5
Age‐at‐neurological‐onset sub‐group, n (%)		
Early infantile (<2 years)	97 (14.5)	85 (14.4)
Late infantile (2 to <6 years)	210 (31.4)	198 (33.6)
Juvenile (6 to <15 years)	198 (29.6)	171 (29.0)
Adolescent/adult (≥15 years)	163 (24.4)	135 (22.9)
Missing	1	1
Age at diagnosis, years		
n	590	590
Mean (SD)	14.5 (13.6)	14.5 (13.6)
Median	10.0	10.0
Minimum, maximum	0.0, 69.8	0.0, 69.8
Diagnosis lag time,^a^ years		
Mean (SD)	4.29 (7.0)	4.29 (7.0)
Miglustat treatment status, n (%)		
Treated with miglustat^b^	384 (57.4)	329 (55.8)
Early infantile (<2 years)	48 (12.5)	41 (12.5)
Late infantile (2 to <6 years)	101 (26.3)	92 (28.0)
Juvenile (6 to <15 years)	116 (30.2)	97 (29.5)
Adolescent/adult (≥15 years)	119 (31.0)	99 (30.1)
Not treated with miglustat	285 (42.6)	261 (44.2)

a Diagnosis lag time: time between onset of neurological manifestations and diagnosis. Please note that some patients have negative values.

b Patient received miglustat at least once.

The Neurological onset group comprised 97 early infantile, 210 late infantile, 198 juvenile, and 163 adolescent/adult patients (Table 1). Of these, 48 (49.5%), 101 (48.1%), 116 (58.6%), and 119 (73.0%) patients in the early infantile, late infantile, juvenile, and adolescent/adult sub‐groups, respectively were ever treated with miglustat. The Diagnosis group comprised 85 early infantile, 198 late infantile, 171 juvenile, and 135 adolescent/adult patients (Table 1). Of these, 41 (48.2%), 92 (46.5%), 97 (56.7%), and 99 (73.3%) patients, respectively, were ever treated with miglustat. A total of 321 patients in the neurological onset group and 286 patients in the diagnosis group were born prior to 1990, of whom 52 were diagnosed with NP‐C prior to 1990.

The homogeneity test of the variance indicated heterogeneity for survival time between the two data sources (the NPC Registry and pooled national cohorts) for both outcomes (*P* < .05).

### 3.2 Survival in the neurological onset group

**FIGURE 1:**
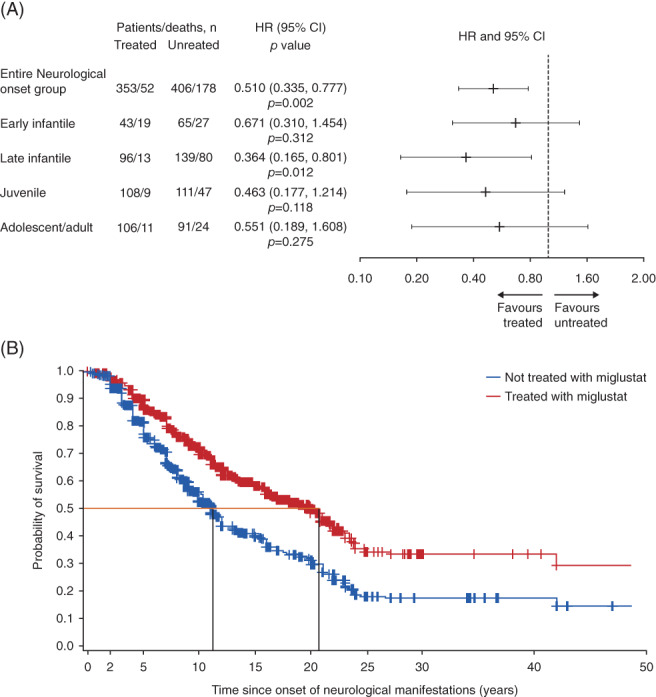
Survival from time of onset of neurological manifestations for miglustat treated vs miglustat‐untreated patients. A, Cox modelling of adjusted HR (extended Cox model covariates: miglustat treatment [time‐varying], country, sex, and age at neurological onset category. Model allows for left‐truncation), entire Neurological onset group and per age‐at‐neurological‐onset sub‐group. B, Adjusted Kaplan–Meier survival curve, entire Neurological onset group. CI, confidence interval; HR, hazard ratio

Miglustat treatment was associated with a significant reduction in the risk of mortality in neurological onset group patients (adjusted HR [95% CI] 0.510 [0.335, 0.777]; *P* < .01) (Figure 1A). The effect was consistent across all four age‐at‐neurological‐onset sub‐groups, with a trend that favoured miglustat treatment; all HRs were between 0.364 (late infantile patients) and 0.671 (early infantile patients). Results were statistically significant for late infantile patients (HR [95% CI] 0.364 [0.165, 0.801]; *P* < .05); statistical significance was not achieved in the remaining age‐at‐neurological‐onset sub‐groups (Figure 1A). Unadjusted HRs demonstrated similar trends (Figure S3A). Adjusted KM curves reflected a reduction in the risk of mortality in patients treated with miglustat vs not treated with miglustat, with ~10 years difference in median survival (Figure 1B). Reduced unadjusted death rates per patient‐years were observed for patients in the miglustat‐treated period vs miglustat‐untreated period in the entire neurological onset group, and the late infantile and juvenile age‐at‐neurological‐onset sub‐groups (Table 2).

**TABLE 2:** Description of crude death rates for miglustat‐treated vs untreated patients

	Patients in the miglustat‐treated period^a^				Patients in the miglustat‐untreated period^b^			
Age‐at‐neurological‐onset sub‐group	No. of patients in the analysis, n	Total PY	Death rate (per 100 PY)	No. (%) of patients who died	No. of patients in the analysis, n	Total PY	Death rate (per 100 PY)	No (%) of patients who died
**Neurological onset group**								
All ages	353	942.9	5.51	52 (14.7)	406	3174.8	5.61	178 (43.8)
Early infantile (<2 years)	43	88.9	21.37	19 (44.2)	65	226.3	11.93	27 (41.5)
Late infantile (2 to <6 years)	96	247.5	5.25	13 (13.5)	139	1016.6	7.87	80 (57.6)
Juvenile (6 to <15 years)	108	320.8	2.81	9 (8.3)	111	1035.8	4.54	47 (42.3)
Adolescent/adult (≥15 years)	106	285.7	3.85	11 (10.4)	91	896.2	2.68	24 (26.4)
**Diagnosis group**								
All ages	307	880.8	5.56	49 (16.0)	353	1846.3	9.42	174 (49.3)
Early infantile (<2 years)	38	82.2	20.67	17 (44.7)	60	161.5	16.72	27 (45.0)
Late infantile (2 to <6 years)	88	239.7	5.42	13 (14.8)	136	706.6	11.04	78 (57.4)
Juvenile (6 to <15 years)	91	298.2	2.68	8 (8.8)	100	647.3	7.26	47 (47.0)
Adolescent/adult (≥15 years)	90	260.6	4.22	11 (12.2)	57	330.8	6.65	22 (38.6)

a Patients received miglustat at any time.

b Patients either never received miglustat or were initially untreated after their onset of neurological manifestations.

Abbreviation: PY, patient‐years.

### 3.3 Survival in the diagnosis group

**FIGURE 2:**
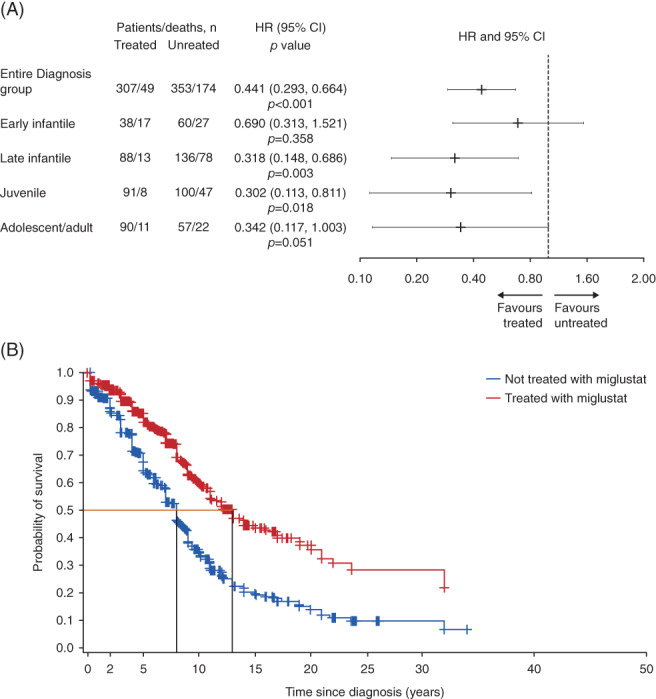
Survival from time of diagnosis for miglustat treated vs miglustat‐untreated patients. A, Cox modelling of adjusted HR (extended Cox model covariates: miglustat treatment [time‐varying], country, sex, and age at neurological onset category. Model allows for left‐truncation), entire Diagnosis group and per age‐at‐neurological‐onset sub‐group. B, Adjusted Kaplan–Meier survival curve, entire Diagnosis group. CI, confidence interval; HR, hazard ratio

Miglustat treatment was associated with a significant reduction in the risk of mortality in diagnosis group patients (HR [95% CI] 0.441 [0.293, 0.664]; *P* < .001) (Figure 2A). The effect was consistent across all four age‐at‐neurological‐onset sub‐groups, favouring miglustat treatment; all HRs were between 0.302 (juvenile patients) and 0.690 (early infantile patients). Results were statistically significant for late infantile (HR [95% CI] 0.318 [0.148, 0.686]; *P* < .01) and juvenile (HR [95% CI] 0.302 [0.113, 0.811]; *P* < .05) patients; statistical significance was not achieved in the remaining age‐at‐neurological‐onset sub‐groups (Figure 2A). Unadjusted HRs demonstrated similar trends (Figure S3B). Adjusted KM curves indicated a reduction in the risk of mortality in patients treated with miglustat vs not treated with miglustat, with approximately 5 years' difference in median survival (Figure 2B). Except for early infantile patients, reduced unadjusted death rates per patient‐years were determined for patients in the miglustat treated period vs miglustat‐untreated period in all age‐at‐neurological‐onset sub‐groups (Table 2).

### 3.4 Miglustat lag time and treatment duration

In the Neurological onset group, late infantile and juvenile patients had shorter median (interquartile range, IQR) miglustat lag time of 3.18 (1.11, 7.32) years and 6.49 (3.19, 13.00) years, respectively vs adolescent/adult patients (8.20 [3.13, 13.67] years). Late infantile and juvenile patients were also treated for longer (median [IQR] duration of treatment 4.02 [2.25, 5.91] years for late infantile, and 4.10 [1.99, 7.00] years for juvenile patients), compared with adolescent/adult patients (median [IQR] duration of treatment 2.89 [1.00, 4.28] years) (Table S2).

Patients of all four age‐at‐neurological‐onset sub‐groups in the Diagnosis group had higher median (IQR) proportions of miglustat treatment compared with patients in the Neurological onset group (Diagnosis group: early infantile, 66.15 [36.71, 80.01]%; late infantile, 78.09 [50.07, 92.37]%; juvenile, 75.61 [50.01, 93.05]%, adolescent/adult, 88.36 [58.19, 97.92]%; neurological onset group: early infantile, 50.82 [28.65, 76.31]%; late infantile, 50.10 [30.48, 80.02]%; juvenile, 38.99 [13.34, 62.22]%, adolescent/adult, 29.32 [9.96; 47.65]%) (data not shown).

### 3.5 Factors influencing the survival model

Time‐varying treatment, age at neurological onset, and country were all found to be significant factors in the survival model (all *P* < .001), whereas sex was not a significant factor (*P* = .142). Additional sensitivity analyses demonstrated that miglustat lag time was (*P* < .001), and data source was not a significant factor (*P* = .658). The influence of the era of diagnosis was not estimable, as the extended Cox model did not converge due to over‐parameterisation.

## 4 DISCUSSION

Several cohort studies and case series indicated beneficial effects of miglustat treatment in patients with NP‐C with regard to disease severity, neurological deterioration, and short‐term (up to 10 years) survival.^12^, ^13^, ^14^, ^15^ The current analysis represents the longest longitudinal analysis of the effect of miglustat treatment on patient survival to date, performed in the largest population of patients with NP‐C. It extends the earlier findings of therapeutic effects of miglustat, demonstrating an increased survival of patients treated with miglustat compared with those who were not treated. Compared with patients not treated with miglustat, median survival of patients treated with miglustat was longer by approximately 10 years from onset of their neurological manifestations, and by ~5 years from their NP‐C diagnosis. Approximately 5‐year difference in median survival between the two analysis groups may be reflective of the observed 4.29‐year mean delay between the onset of neurological manifestations and NP‐C diagnosis, with the KM curves also demonstrating a clear trend for longer survival with miglustat treatment.

The increased survival of patients treated with miglustat was consistent across all four age‐at‐neurological‐onset sub‐groups, significantly for late infantile patients when analysed from onset of neurological manifestations, and for late infantile and juvenile patients when analysed from their NP‐C diagnosis. Low numbers of patients and different treatment periods likely contributed to non‐significant results in the remaining sub‐groups. The effect of miglustat treatment was expectedly least pronounced for patients with the early infantile neurological onset, in whom NP‐C is known to progress more rapidly and has potentially less treatment responsiveness. There is a lag time of 6‐12 months between the initiation of miglustat treatment and observable beneficial clinical effects^10^, ^20^; the disease in early infantile patients may advance too rapidly for miglustat to provide much benefit to their survival. The clinical picture of adult‐onset NP‐C is often subtle and non‐specific for several years.^20^, ^21^ The longer miglustat lag time observed for adult/adolescent patients may also explain the smaller benefit of miglustat treatment in this age‐at‐neurological‐onset sub‐group. Diagnosis of NP‐C is often delayed for many years with the disease becoming very advanced, limiting the effectiveness of subsequent miglustat treatment. Furthermore, in patients with adult neurological onset NP‐C is more slowly progressive compared with other neurological onset ages, and although there may be a beneficial effect of miglustat, it may be more difficult to detect over the observation period.

The magnitude of effect of miglustat treatment on patient survival was more pronounced in the diagnosis group vs the Neurological onset group, with more age‐at‐neurological‐onset sub‐groups exhibiting statistically significant findings and lower hazard ratios. This difference can be explained by overall higher proportions, and longer duration, of miglustat treatment of patients in the diagnosis group vs the neurological onset group. Furthermore, the exact date of first neurological manifestation onset may be less accurate due to initially subtle neurological signs being missed entirely, or inaccurately recalled. It is important to mention that although considered a more accurate measure in the survival analysis, the timing of NP‐C diagnosis can also occur at different stages of the disease course for each patient.

Death rates per patient‐years and proportions of patients who died were lower for miglustat treated patients vs not treated, except for the early infantile patients from the Diagnosis group, and for the early infantile and adolescent/adult patients from the Neurological onset group. Because of the small number of patients, and consequently less number of patient‐years within each age‐at‐neurological‐onset sub‐group, variability between individuals had a large impact on the overall death rates. The reported crude death rates did not account for covariates, which also contributed to the variability of the results. The overall number of patient‐years was lower in the miglustat treated period compared with untreated, which resulted in a more pronounced variability in data of the treated population; a trend of generally lower death rates in treated patients was still observed.

Heterogeneity in the data sources and patient characteristics was expected. Variable medical practices between and within countries and time periods, as well as differences in the size and protocol design of the NPC Registry compared with the national cohorts, may have confounded the above findings. Several of these confounders were investigated in separate sensitivity analyses. Age at onset of neurological manifestations is widely recognised as a predictor for disease severity, progression, and survival^2^, ^4^, ^5^, ^6^, ^7^ and was confirmed to be a significant factor in the model. Other factors that were found to influence the survival model were country (reflecting differences in access to care), practice, populations, availability of miglustat treatment and miglustat lag time. Further limitations of the analysis included difficulty in adjusting for disease severity (data on progression of disability not collected, no genotype information available), and recall bias (US cohort).

That miglustat has contributed to improved survival of patients with NP‐C is reinforced by the data of a recent “crowd‐sourced” analysis of age and year of death of more than 300 patients with NP‐C who died between 1968 and 2018, based on information posted on disease support group website memorial walls.^22^ This analysis found no significant change in overall survival over the last decades suggesting that supportive medical care has not impacted survival in the recent past.

In conclusion, this study is the first exploratory analysis conducted in the largest pool of observational data for patients with NP‐C to date, treated in a real‐world setting and with a broad geographical representation. Despite the limitations of the data that urge cautious interpretation, the findings are consistent with a beneficial effect of miglustat on survival in patients with NP‐C.

## CONFLICT OF INTEREST

Marc Patterson has received travel expenses and/or speaker honoraria and/or consultancy fees and/or research funding from Actelion Pharmaceuticals Ltd., Amicus, IntraBio, Novartis, Orphazyme, Shire and Vtesse; he owns stock in IntraBio. Roberto Giugliani has received travel grants, and/or speaker honoraria and/or consultancy fees from Actelion Pharmaceuticals Ltd., Amicus, Armagen, BioMarin, Chiesi, GC Pharma, Inventiva, JCR, Lysogene, RegenxBio, Sanofi‐Genzyme, Shire, Sobi, and Ultragenyx. Benedicte Héron has received travel expenses from, and attended meetings funded and organised by Actelion Pharmaceuticals Ltd., and has received presentation honoraria from Actelion Pharmaceuticals Ltd. She is principal investigator in France for clinical trials conducted by Orphazyme and Vtesse/Mallinckrodt on NPC disease. Patrick Moneuse is an employee of Actelion Pharmaceuticals Ltd. Olivier Morand was an employee of Actelion Pharmaceuticals Ltd. at the time of the study. Daniel Rosenberg is an employee of Actelion Pharmaceuticals Ltd. Barbara Schwierin was an employee of Actelion Pharmaceuticals Ltd. at the time of the study. William Sherman Garver, Jackie Imrie, Helena Jahnova, Francis John Meaney, Yann Nadjar and Marie Vanier declare that they have no conflict of interest.

## AUTHOR CONTRIBUTIONS

Patrick Moneuse, Olivier Morand, Daniel Rosenberg, Barbara Schwierin participated in the conception and design of the work as well as in the analysis or interpretation of the data. All authors participated in the acquisition/collection of data, and in the analysis or interpretation of the data. All authors critically reviewed the manuscript and approved it for submission.

## Supporting information


**Supplementary Figure 1** Miglustat treatment exposure groups and periods

**Supplementary Figure 2** Patient disposition

**Supplementary Figure 3** Cox modelling of unadjusted HR* for miglustat treated vs miglustat‐untreated patients, entire group and per age‐at‐neurological‐onset sub‐group. (A) Survival from time of onset of neurological manifestations. (B) Survival from time of diagnosis.

**Appendix** **S1**: Supporting information

## Data Availability

Data are available upon receipt of reasonable request.
