# Supplementary figures and images for: Long‐term survival outcomes of patients with Niemann‐Pick disease type C receiving miglustat treatment: A large retrospective observational study
Source: J Inherit Metab Dis. 2020 May 8;43(5):1060–9. doi: 10.1002/jimd.12245 (PMC7540716; doi:10.1002/jimd.12245)

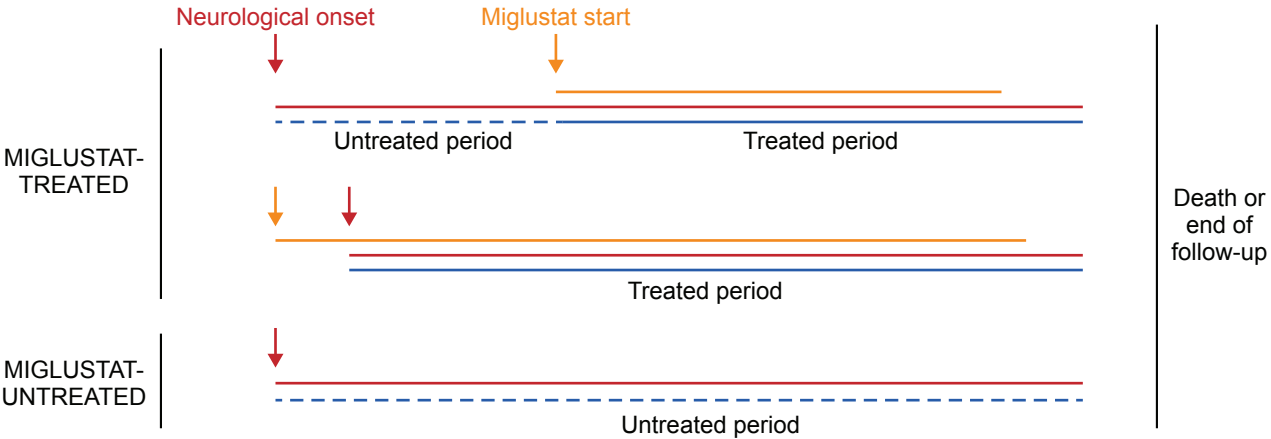

Supplement: Supplementary file 1 — Supplementary Figure 1 Miglustat treatment exposure groups and periods [file JIMD-43-1060-s001.pdf]

**A**

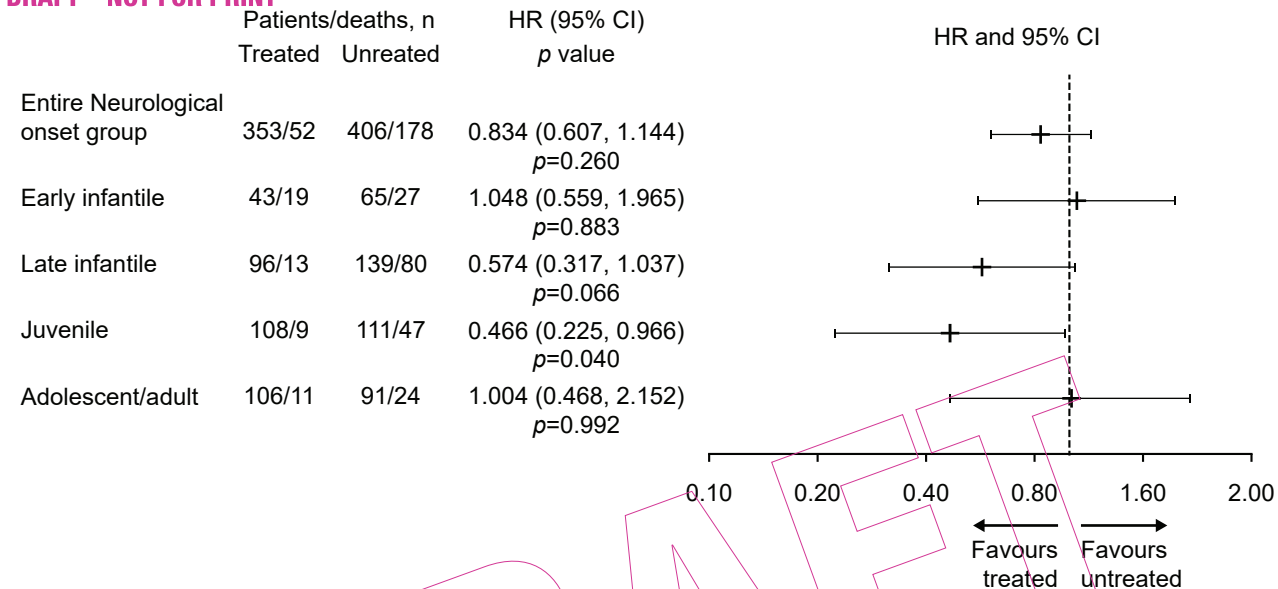

**B**

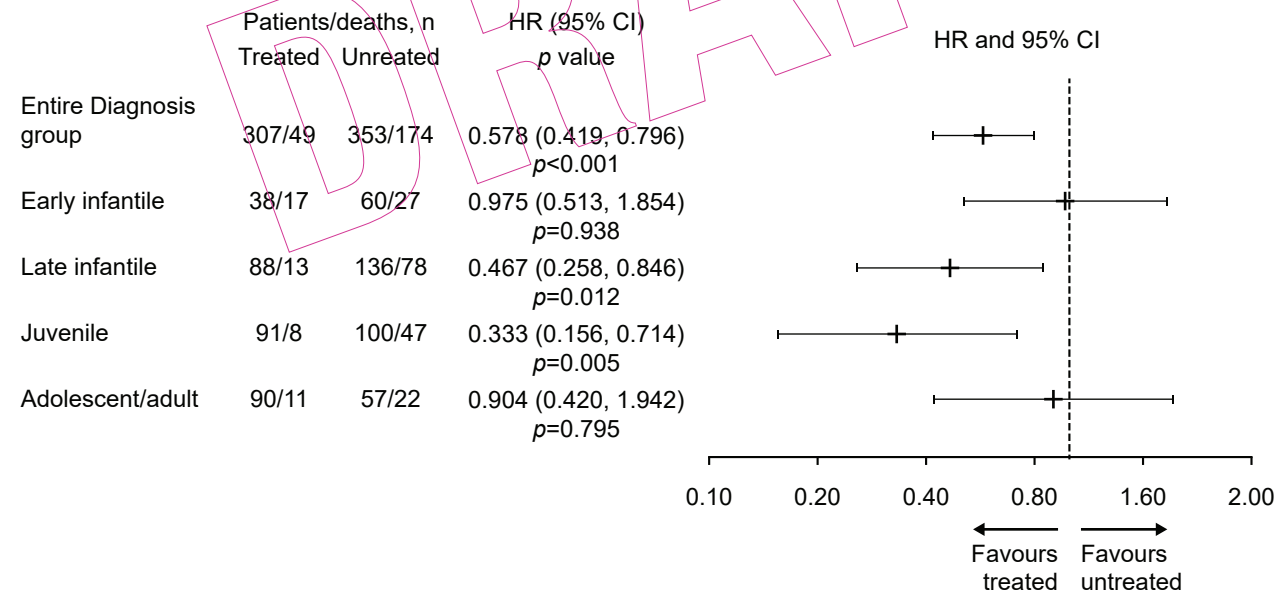

Supplement: Supplementary file 3 — Supplementary Figure 3 Cox modelling of unadjusted HR* for miglustat treated vs miglustat‐untreated patients, entire group and per age‐at‐neurological‐onset sub‐group. (A) Survival from time of onset of neurological manifestations. (B) Survival from time of diagnosis. [file JIMD-43-1060-s003.pdf]
