# Supplementary material for: Long‐term survival outcomes of patients with Niemann‐Pick disease type C receiving miglustat treatment: A large retrospective observational study
Source: J Inherit Metab Dis. 2020 May 8;43(5):1060–9. doi: 10.1002/jimd.12245 (PMC7540716; doi:10.1002/jimd.12245)
Supplement: Supplementary file 2 — Supplementary Figure 2 Patient disposition [file JIMD-43-1060-s002.pdf]

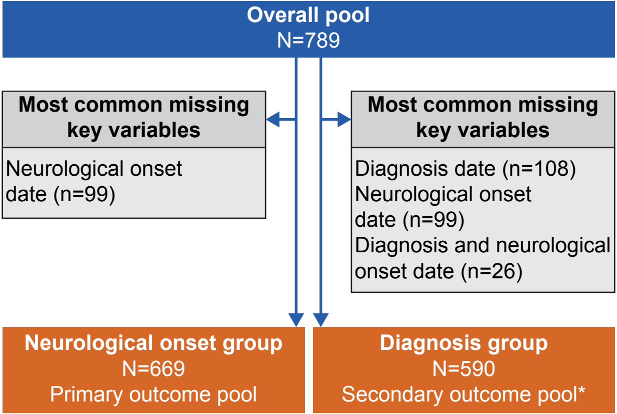
Overall pool
N=789
Most common missing key variables
Neurological onset date (n=99)
Most common missing key variables
Diagnosis date (n=108)
Neurological onset date (n=99)
Diagnosis and neurological onset date (n=26)
Neurological onset group
N=669
Primary outcome pool
Diagnosis group
N=590
Secondary outcome pool*
