# Supplementary material for: Long‐term survival outcomes of patients with Niemann‐Pick disease type C receiving miglustat treatment: A large retrospective observational study
Source: J Inherit Metab Dis. 2020 May 8;43(5):1060–9. doi: 10.1002/jimd.12245 (PMC7540716; doi:10.1002/jimd.12245)
Supplement: Supplementary file 4 — Appendix S1: Supporting information [file JIMD-43-1060-s004.docx]

***Supplementary information***

**Supplementary Table 1. Patient data sources**

| **NPC Registry (21 countries)** | **N** | **Data source** | **Study population** |
| --- | --- | --- | --- |
| Australia, Austria, Brazil, Bulgaria, Canada, Czech Republic, Denmark, France, Germany, Greece, Italy, the Netherlands, Norway, Poland, Portugal, Slovak Republic, Slovenia, Spain, Sweden, Switzerland, UK | 414 | Prospective (Actelion) | Incident & prevalent patients (2009–2016) |
| **National cohorts** | **N** | **Data source** | **Study population** |
| Brazil | 51 | Medical chart review | Incident patients (1991–2015) |
| Czech Republic | 57 | Medical chart review | Incident & prevalent patients (1975–2012) |
| France | 136 | Medical chart review | Incident patients (1990–2014) |
| UK | 146 | Medical chart review | Incident & prevalent patients (1999–2011) |
| USA | 88 | Patient/family reported (questionnaire) | Incident & prevalent patients (1975–2002) |

**Supplementary Figure 1. Miglustat treatment exposure groups and periods**


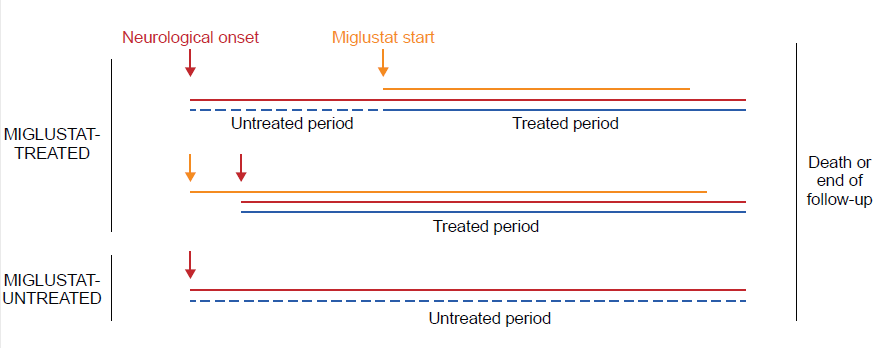


Untreated period is defined as a period when a patient is not treated with miglustat; treated period is defined as a period when a patient is treated with miglustat. A patient is considered treated if he/she were ever treated with miglustat, and not-treated if he/she were never treated with miglustat. As soon as a patient has started miglustat, he/she is considered treated until death or last follow-up. Patients had a different follow-up time.

**Supplementary Figure 2. Patient disposition**


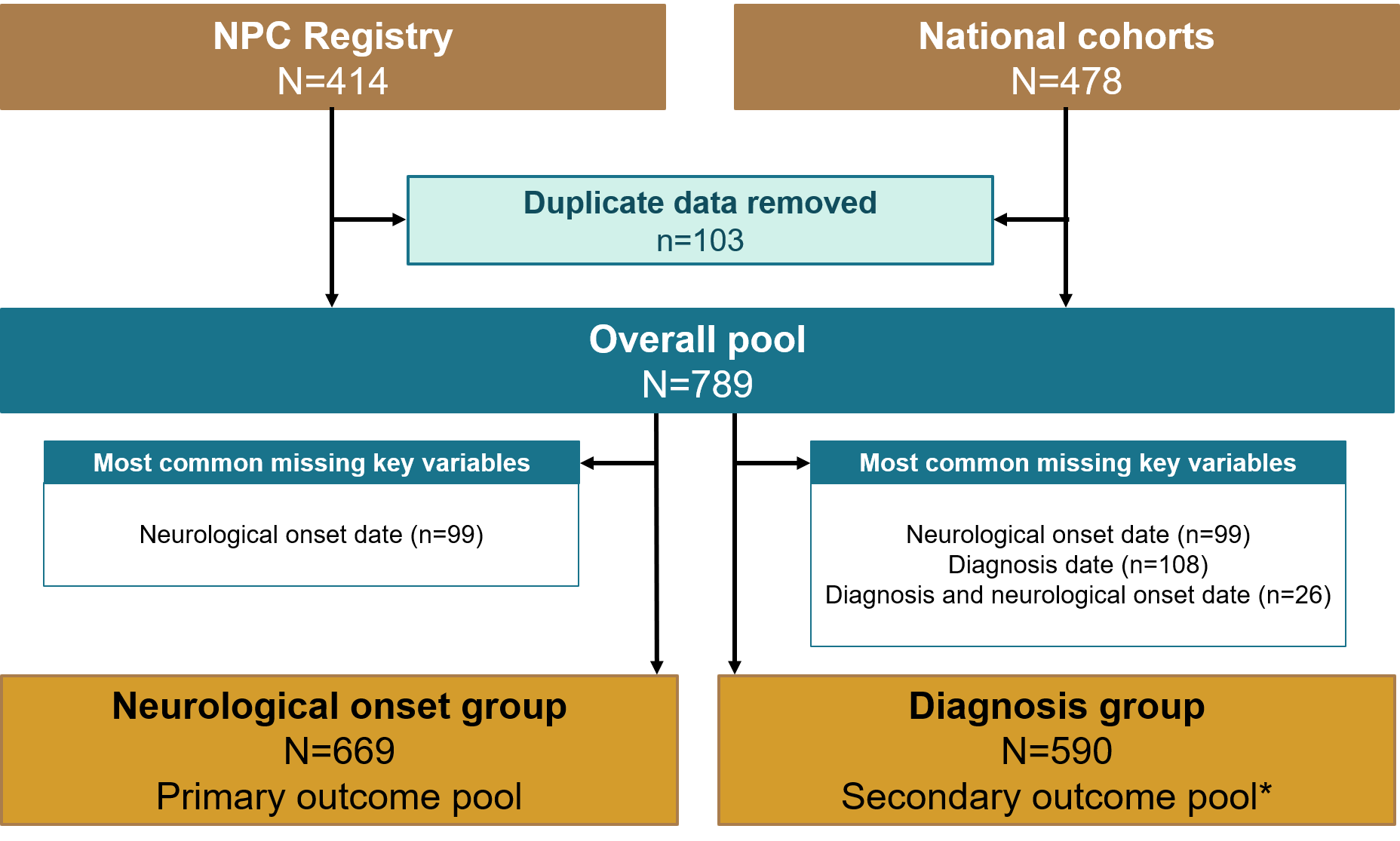


Note that the same overall pool is used to generate the primary and secondary outcome pools, by excluding different sets of patients for whom data for the key variables were missing. *Patients in the secondary outcome pool may have more than one of the key variables missing.

**Supplementary Figure 3. Cox modelling of unadjusted HR* for miglustat treated *versus* miglustat-untreated patients, entire group and per age-at-neurological-onset sub-group. (A) Survival from time of onset of neurological manifestations. (B) Survival from time of diagnosis.**


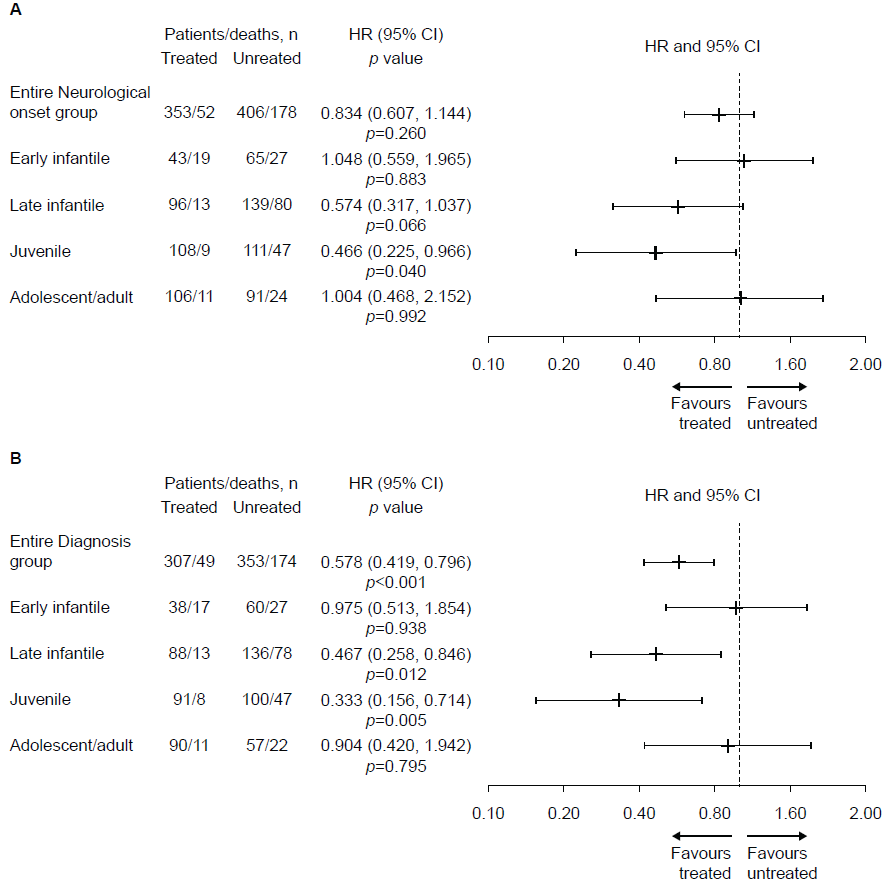


*****Extended Cox model covariates: miglustat treatment (time-varying), country, sex, and age at neurological onset category. Model allows for left-truncation.

CI, confidence interval; HR, hazard ratio

**Supplementary Table 2. Miglustat lag time and duration of treatment for patients in the Neurological onset group**

| **Age-at-neurological-onset sub-group** | **Age at neurological onset,** years (median [IQR]) | **Age at miglustat treatment start,** years (median [IQR]) | **Miglustat lag time**, years (median [IQR]) | **Duration of treatment,** years (median [IQR]) |
| --- | --- | --- | --- | --- |
| **Early infantile**  (<2 years) | **1.00**  (0.40, 1.23) | **3.34**  (1.99, 4.52) | **2.13**  (0.95, 3.44) | **2.41**  (1.37, 4.42) |
| **Late infantile**  (2 to <6 years) | **4.00**  (2.77, 5.23) | **7.21**  (4.55, 11.19) | **3.18**  (1.11, 7.32) | **4.02**  (2.25, 5.91) |
| **Juvenile**  (6 to <15 years) | **10.00**  (8.00, 12.00) | **16.89**  (13.04, 22.21) | **6.49**  (3.19, 13.00) | **4.10**  (1.99, 7.00) |
| **Adolescent/adult**  (≥15 years) | **23.17**  (17.61, 31.38) | **33.05**  (26.18, 42.64) | **8.20**  (3.13, 13.67) | **2.89**  (1.00, 4.28) |

IQR, interquartile range
